# Treatment patterns and survival analysis in patients with unresectable stage III EGFR-mutated non-small cell lung cancer

**DOI:** 10.18632/aging.205425

**Published:** 2024-01-11

**Authors:** Huan-Wei Liang, Yang Liu, Xin-Bin Pan

**Affiliations:** 1 Department of Radiation Oncology, Guangxi Medical University Cancer Hospital, Nanning 530021, Guangxi, P.R. China

**Keywords:** non-small cell lung cancer, NSCLC, epidermal growth factor receptor, EGFR, tyrosine kinase inhibitor

## Abstract

Purpose: To investigate the treatment patterns and survival outcomes in patients with unresectable Stage III EGFR-mutated non-small cell lung cancer (NSCLC).

Materials and methods: A retrospective analysis was conducted on patients with unresectable Stage III EGFR-mutated NSCLC spanning from 2012 to 2022. Treatment patterns were outlined, and survival comparisons between different treatment groups were performed using Kaplan-Meier methods.

Results: A total of 88 patients were included: 62.5% received TKI alone, 26.1% received TKI+chemotherapy, 4.5% received radiotherapy, 4.5% participated in clinical trials, and 2.4% received TKI+antiangiogenic drugs. Prior to propensity score matching, TKI+chemotherapy and TKI alone groups demonstrated similar progression-free survival (hazard ratio [HR] = 1.56, 95% confidence interval [CI]: 0.87-2.80; P = 0.134), overall survival (HR = 1.12, 95% CI: 0.59-2.13; P = 0.733), and locoregional-free survival (HR = 1.46; 95% CI: 0.75-2.81; P = 0.267). However, TKI+chemotherapy showed reduced distant metastasis-free survival compared to TKI alone (HR = 2.39, 95% CI: 1.11-5.18; P = 0.022). After propensity score matching, no significant differences were observed in progression-free survival (P = 0.435), overall survival (P = 0.205), locoregional-free survival (P = 0.706), and distant metastasis-free survival (P = 0.171) between the TKI+chemotherapy and TKI alone groups.

Conclusions: The addition of chemotherapy to TKI did not enhance survival outcomes compared to TKI monotherapy in patients with unresectable Stage III EGFR-mutated NSCLC.

## INTRODUCTION

Lung cancer is a major contributor to cancer-related deaths worldwide [1]. Specifically, non-Small Cell Lung Cancer (NSCLC) accounts for approximately 85.0% of all new lung cancer cases, [2] with roughly 30% of these NSCLC patients being diagnosed with stage III disease [3, 4]. Stage III NSCLC encompasses a diverse range of diseases, [5] resulting in a variety of treatment approaches [6–11]. Currently, the standard treatment for unresectable epidermal growth factor receptor (EGFR) wild-type cases is concurrent chemoradiotherapy (CCRT) plus adjuvant durvalumab [12–15]. However, for EGFR-mutated patients, adjuvant durvalumab is not recommended, and CCRT remains the standard care.

Despite its status as the standard treatment, several studies have reported that CCRT may lead to poorer survival outcomes in EGFR-mutated patients [16, 17]. Conversely, in clinical practice, EGFR-tyrosine kinase inhibitor (TKI) is often recommended for stage III cases based on survival data from trials designed for stage IV EGFR-mutated NSCLC [18–24]. To date, the optimal treatment strategy for unresectable stage III EGFR-mutated NSCLC patients remains unclear. Therefore, this study aims to investigate the treatment patterns and survival rates in this specific patient population.

## RESULTS

### Patient characteristics

**Figure 1:**
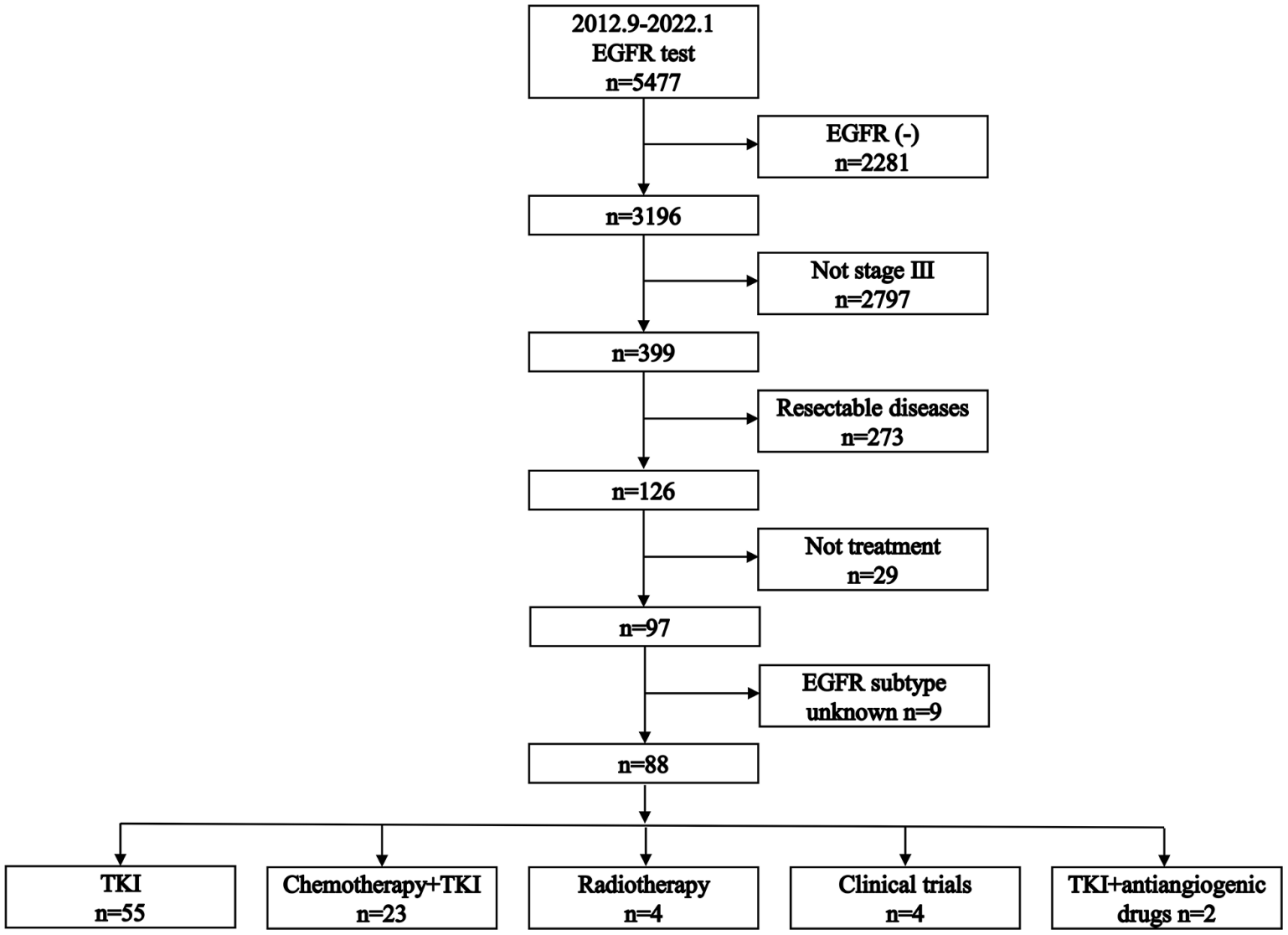
Flowchart of patient selection process.

The selection process of patients is visually represented in Figure 1. Out of the 5,477 patients tested for EGFR status, 88 were deemed eligible for the study. A comprehensive summary of these patients’ characteristics can be found in Table 1. Notably, the median follow-up time for these patients was 19 months, with an interquartile range of 11-29 months.

**Table 1:** Patient characteristics.

**Characteristics**	**Total (n=88)**
Age (year) median (IQR)	63 (54-70)
Sex	
Male	42 (47.7%)
Female	46 (52.3%)
Smoking status	
Current smoker	4 (4.5%)
Former smoker	19 (21.6%)
Never smoker	65 (73.9%)
ECOG	
0	44 (50.0%)
1	43 (48.9%)
≥2	1 (1.1%)
T stage	
T1	20 (22.7%)
T2	32 (36.4%)
T3	15 (17.0%)
T4	21 (23.9%)
N stage	
N0	2 (2.2%)
N1	3 (3.4%)
N2	32 (36.4%)
N3	51 (58.0%)
AJCC stage	
IIIa	22 (25.0%)
IIIb	51 (58.0%)
IIIc	15 (17.0%)
EGFR mutation	
Exon 19 deletion	49 (55.7%)
L858R mutation	33 (37.5%)
Other	6 (6.8%)

IQR, interquartile range; ECOG, Eastern Cooperative Oncology Group; AJCC, American Joint Committee on Cancer; EGFR, epidermal growth factor receptor.

### Treatment patterns

**Figure 2:**
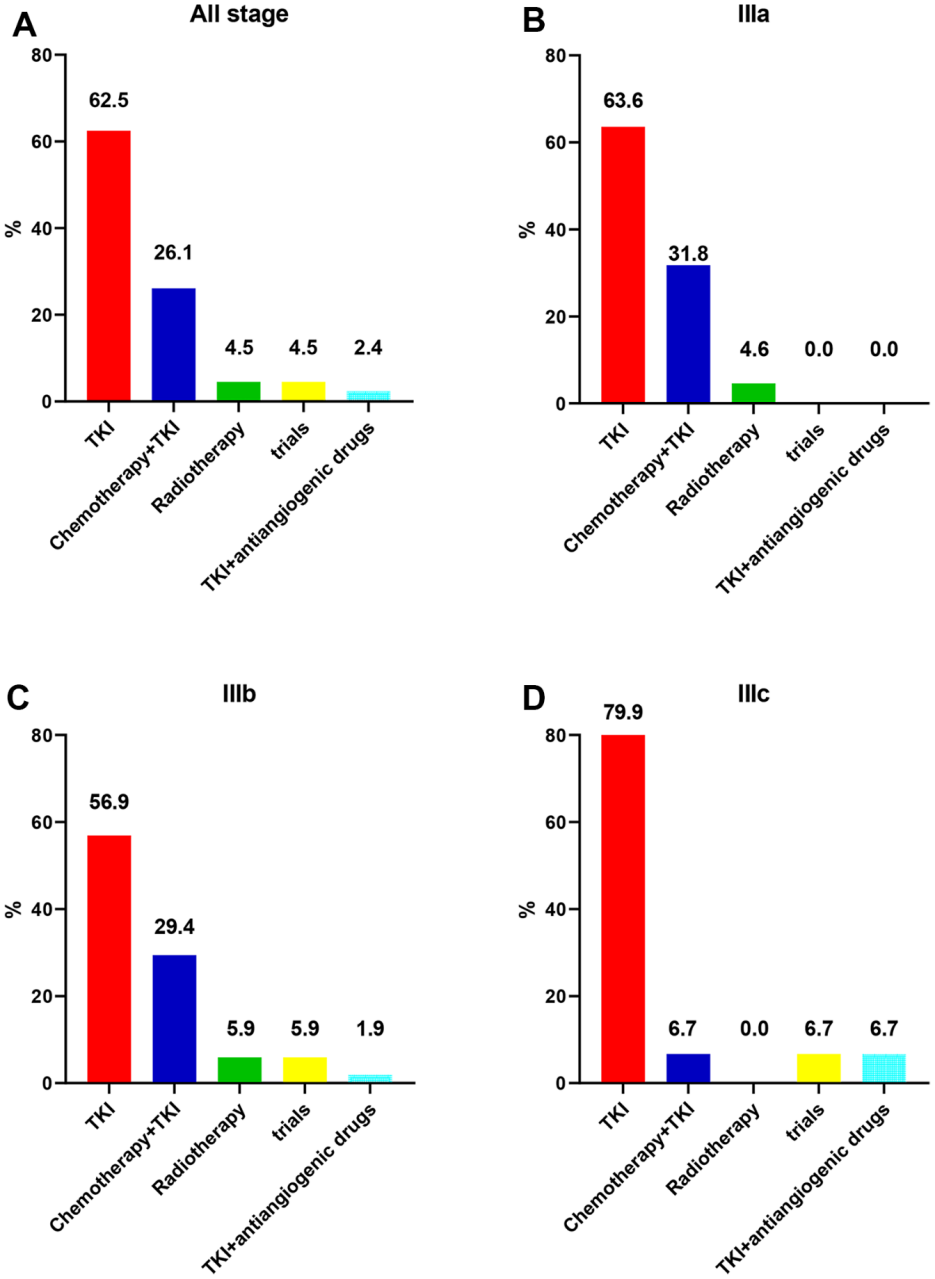
**Initial treatment patterns for unresectable stage III EGFR-mutated non-small cell lung cancer.** (**A**) Stage III. (**B**) Stage IIIa. (**C**) Stage IIIb. (**D**) Stage IIIc.

The various treatment approaches administered to the patients are detailed in Figure 2. Overall, 62.5% of the patients received TKI alone, while 26.1% received TKI+chemotherapy. Radiotherapy was administered to 4.5% of the patients, 4.5% participated in clinical trials, and 2.4% received a regimen consisting of TKI+antiangiogenic drugs.

Due to the small numbers of patients in the radiotherapy, antiangiogenic therapy, and clinical trials subgroups, the survival analysis primarily compared the outcomes between the TKI alone group and the TKI+chemotherapy group. The specific characteristics of patients in these two subgroups before and after PSM are detailed in Table 2.

**Table 2:** Patient characteristics between TKI alone and TKI+chemotherapy groups.

	**The unmatched cohort**		***P***	**The PSM cohort**		***P***
	**TKI alone (n=55)**	**TKI+chemotherapy (n=23)**		**TKI alone (n=20)**	**TKI+chemotherapy (n=20)**	
Age (year)			0.019			0.999
≤63	23 (41.8%)	17 (73.9%)		13 (65.0%)	14 (70.0%)	
>63	32 (58.2%)	6 (26.1%)		7 (35.0%)	6 (30.0%)	
Sex			0.005			0.740
Male	20 (36.4%)	17 (73.9%)		12 (60.0%)	14 (70.0%)	
Female	35 (63.6%)	6 (26.1%)		8 (40.0%)	6 (30.0%)	
Smoking status			0.064			0.780
Current smoker	1 (1.8%)	3 (13.0%)		1 (5.0%)	3 (15.0%)	
Former smoker	10 (18.2%)	6 (26.1%)		5 (25.0%)	4 (20.0%)	
Never smoker	44 (80.0%)	14 (60.9%)		14 (70.0%)	13 (65.0%)	
ECOG			0.954			0.999
0	29 (52.7%)	13 (56.5%)		11 (55.0%)	11 (55.0%)	
1	26 (47.3%)	10 (43.5%)		9 (45.0%)	9 (45.0%)	
T stage			0.012			0.382
T1	9 (16.4%)	9 (39.1%)		6 (30.0%)	9 (45.0%)	
T2	19 (34.5%)	10 (43.5%)		12 (60.0%)	7 (35.0%)	
T3	13 (23.6%)	0 (0.0%)		/	/	
T4	14 (25.5%)	4 (17.4%)		2 (10.0%)	4 (20.0%)	
N stage			0.821			0.341
N0	2 (3.7%)	0 (0.0%)		/	/	
N1	1 (1.8%)	1 (4.3%)		0 (0.0%)	1 (5.0%)	
N2	22 (40.0%)	8 (34.8%)		11 (55.0%)	7 (35.0%)	
N3	30 (54.5%)	14 (60.9%)		9 (45.0%)	12 (60.0%)	
AJCC stage			0.181			0.514
IIIa	14 (25.5%)	7 (30.4%)		9 (45.0%)	6 (30.0%)	
IIIb	29 (52.7%)	15 (65.2%)		11 (55.0%)	13 (65.0%)	
IIIc	12 (21.8%)	1 (4.4%)		0 (0.0%)	1 (5.0%)	
EGFR mutation			0.373			0.715
Exon 19 deletion	28 (50.9%)	16 (69.6%)		14 (70.0%)	16 (80.0%)	
L858R mutation	22 (40.0%)	6 (26.1%)		6 (30.0%)	4 (20.0%)	
Other	5 (9.1%)	1 (4.3%)		/	/	

### PFS

**Figure 3:**
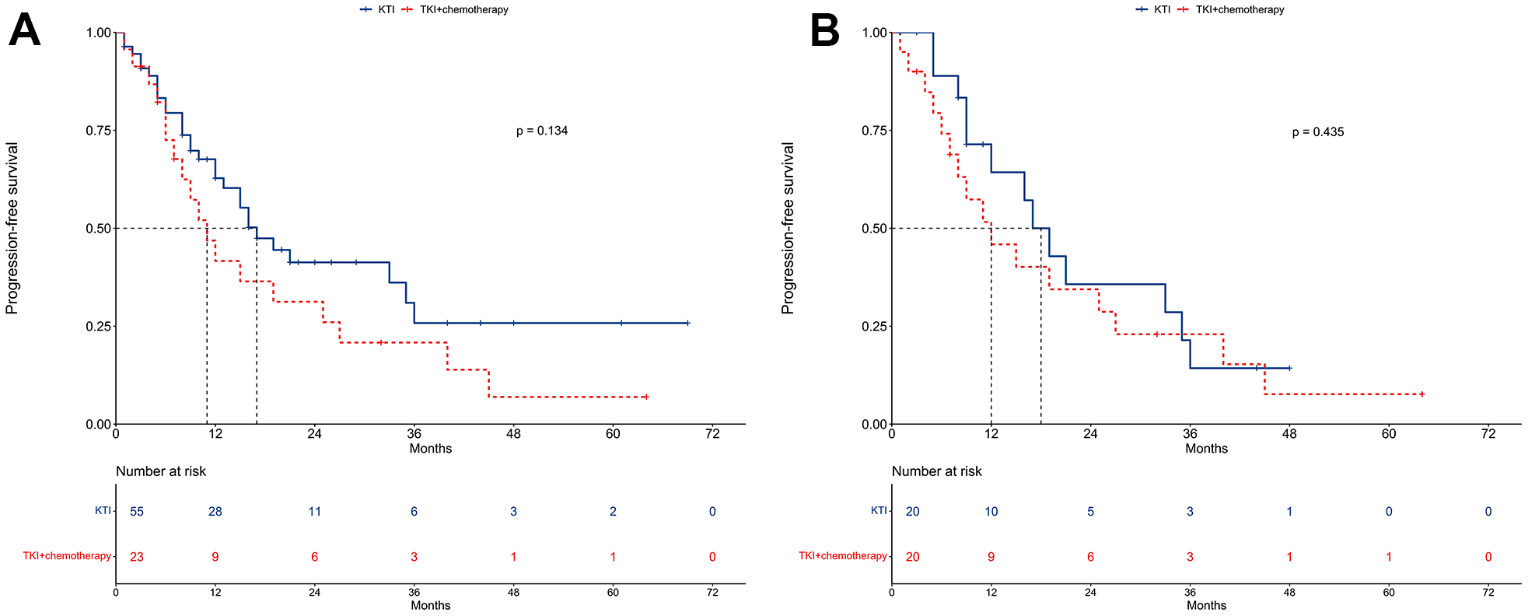
**Progression-free survival between TKI alone and TKI+chemotherapy groups.** (**A**) The unmatched cohort. (**B**) The propensity score matching cohort.

In the unmatched cohort, the median PFS for the TKI alone group was 17 months, compared to 11 months for the TKI plus chemotherapy group (Figure 3A). The 1-year PFS rates were 62.8% and 41.6%, respectively, for the two groups. Similarly, the 2-year PFS rates were 41.3% and 31.2%, respectively. The univariable regression analysis revealed no statistically significant difference in PFS between the two groups (HR = 1.56, 95% CI: 0.87-2.80; P = 0.134; Table 3). Furthermore, multivariable Cox regression analysis confirmed that TKI+chemotherapy was not an independent prognostic factor for PFS (HR = 1.47, 95% CI: 0.73-2.97; P = 0.276; Table 3).

**Table 3:** Univariable and multivariable Cox regressions of progression-free survival.

	**Univariable analysis**			**Multivariable analysis**		
	**HR**	**95% CI**	***P***	**HR**	**95% CI**	***P***
Age						
≤63	reference					
>63	0.54	0.30-0.97	0.043	0.54	0.25-1.13	0.099
Sex						
Male	reference					
Female	0.87	0.49-1.54	0.633	0.67	0.30-1.46	0.306
Smoking status						
Never smoker	reference					
Former smoker	0.77	0.36-1.66	0.510	0.54	0.18-1.53	0.235
Current smoker	2.21	0.52-9.41	0.284	1.83	0.08-3.66	0.534
ECOG						
0	reference					
1	0.66	0.36-1.18	0.158	0.58	0.26-1.25	0.160
T stage						
T1	reference					
T2	1.31	0.61-2.83	0.493	1.54	0.69-3.53	0.295
T3	1.14	0.54-3.00	0.796			
T4	1.87	0.79-4.43	0.155			
N stage						
N0	reference					
N1	4.17	0.38-46.25	0.245	7.96	0.49-142.27	0.144
N2	1.52	0.20-11.43	0.684			
N3	1.73	0.23-12.80	0.591			
AJCC stage						
IIIa	reference					
IIIb	1.10	0.57-2.11	0.775			
IIIc	1.47	0.62-3.53	0.384			
EGFR mutation						
Exon 19 deletion	reference					
L858R mutation	0.83	0.44-1.55	0.549	0.99	0.48-2.13	0.977
Other	0.53	0.13-2.24	0.298	0.54	0.10-2.86	0.468
Treatments						
TKI	reference					
TKI+chemotherapy	1.56	0.87-2.80	0.134	1.47	0.73-2.97	0.276

ECOG, Eastern Cooperative Oncology Group; AJCC, American Joint Committee on Cancer; TKI, tyrosine kinase inhibitor; EGFR, epidermal growth factor receptor; HR, hazard ratio; CI, confidence interval.

In the PSM cohort, the median PFS for the TKI alone group and the TKI+chemotherapy group was 18 months and 12 months, respectively (Figure 3B). The corresponding 1-year PFS rates were 64.3% and 45.9%, while the 2-year PFS rates were 35.7% and 34.4%.

### OS

**Figure 4:**
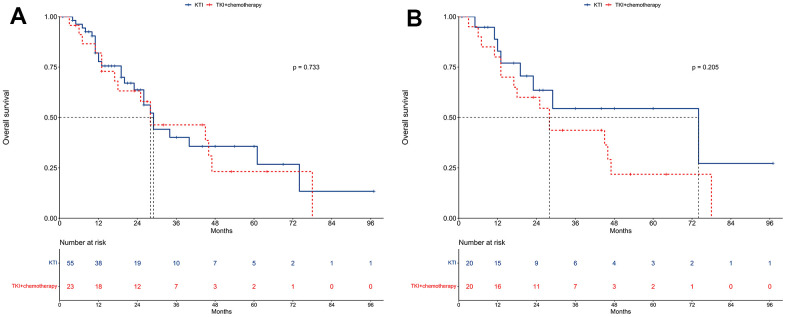
**Overall survival between TKI alone and TKI+chemotherapy groups.** (**A**) The unmatched cohort. (**B**) The propensity score matching cohort.

In the unmatched cohort, the median OS was 29 months for the TKI alone group and 28 months for the TKI+chemotherapy group (Figure 4A). The 1-year OS rates were 77.7% and 82.0%, respectively, for the two groups, while the 2-year OS rates were 63.7% and 63.2%. According to the univariable regression analysis, no significant difference in OS was observed between the two groups (HR = 1.12, 95% CI: 0.59-2.13; P = 0.733; Table 4). Furthermore, the multivariable Cox regression analysis also supported this finding by showing that TKI+chemotherapy was not an independent prognostic factor for OS (HR = 1.57, 95% CI: 0.69-3.54; P = 0.280; Table 4).

**Table 4:** Univariable and multivariable Cox regressions of overall survival.

	**Univariable analysis**			**Multivariable analysis**		
	**HR**	**95% CI**	***P***	**HR**	**95% CI**	***P***
Age						
≤63	reference					
>63	1.35	0.72-2.55	0.347	2.09	0.93-4.68	0.073
Sex						
Male	reference					
Female	1.13	0.59-2.14	0.715	1.38	0.56-2.03	0.477
Smoking status						
Never smoker	reference					
Former smoker	0.72	0.28-1.86	0.496	0.46	0.10-2.05	0.307
Current smoker	6.62	2.15-20.41	0.001	10.19	2.15-48.33	0.003
ECOG						
0	reference					
1	0.76	0.39-1.45	0.400	0.88	0.38-2.03	0.761
T stage						
T1	reference					
T2	0.85	0.35-2.10	0.729	0.73	0.28-1.93	0.526
T3	1.53	0.55-4.29	0.416	21.60	1.78-262.79	0.016
T4	1.93	0.76-4.87	0.164	24.34	1.48-400.73	0.026
N stage						
N0	reference					
N1	1.42	0.09-22.97	0.804	0.18	0.01-4.34	0.287
N2	0.73	0.09-5.57	0.758	4.68	0.17-126.98	0.359
N3	0.85	0.11-6.42	0.878			
AJCC stage						
IIIa	reference					
IIIb	1.86	0.86-4.03	0.114	0.14	0.01-2.43	0.177
IIIc	1.71	0.61-4.94	0.302	0.02	0.01-1.18	0.058
EGFR mutation						
Exon 19 deletion	reference					
L858R mutation	1.04	0.53-2.04	0.912	1.24	0.52-2.95	0.630
Other	0.41	0.06-3.00	0.390	0.24	0.03-2.35	0.222
Treatments						
TKI	reference					
TKI+chemotherapy	1.12	0.59-2.13	0.733	1.57	0.69-3.54	0.280

ECOG, Eastern Cooperative Oncology Group; AJCC, American Joint Committee on Cancer; TKI, tyrosine kinase inhibitor; EGFR, epidermal growth factor receptor; HR, hazard ratio; CI, confidence interval.

In the PSM cohort, the median OS was 74 months for the TKI alone group and 28 months for the TKI+chemotherapy group (Figure 4B). The 1-year OS was 82.9% and 80.0%, respectively, for these two groups, while the 2-year OS was 63.5% and 60.0%, respectively.

### LRFS

**Figure 5:**
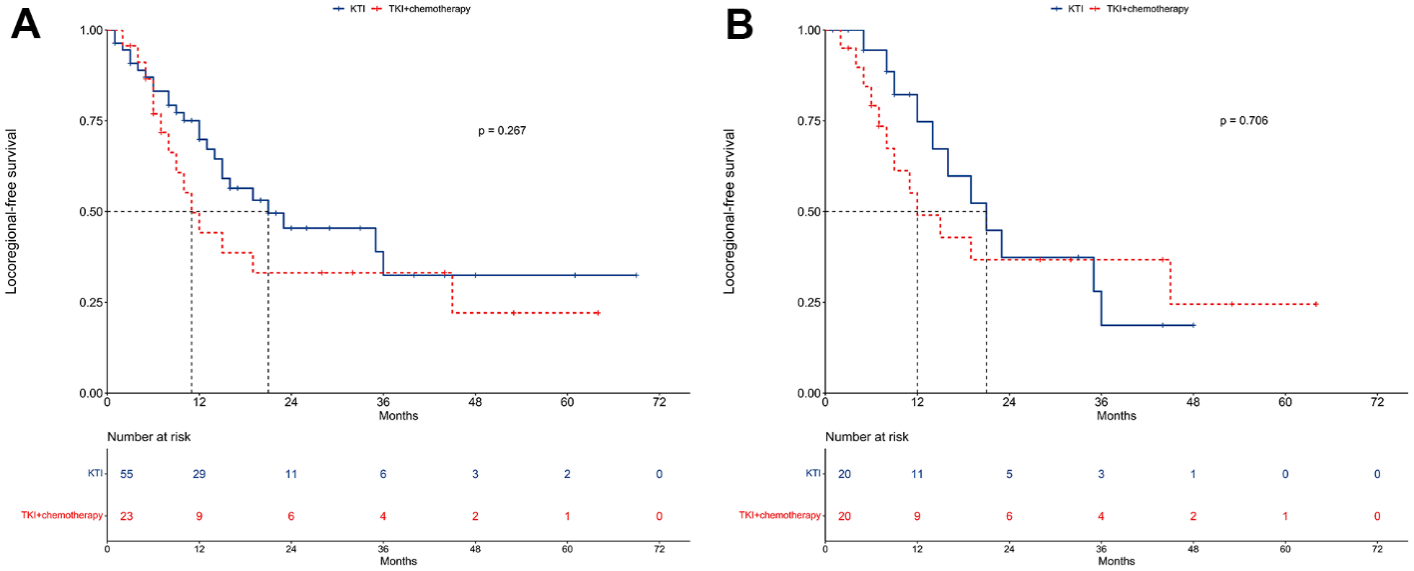
**Locoregional-free survival between TKI alone and TKI+chemotherapy groups.** (**A**) The unmatched cohort. (**B**) The propensity score matching cohort.

In the unmatched cohort, the median LRFS for the TKI alone group was 21 months, compared to 11 months for the TKI+chemotherapy group (Figure 5A). The 1-year LRFS rates were 69.9% and 44.2%, and the 2-year LRFS rates were 45.4% and 33.1%, respectively for the two groups. The univariable regression analysis did not reveal any significant differences in LRFS between the two treatment approaches (HR = 1.46, 95% CI: 0.75-2.81; P = 0.267; Table 5). Additionally, the multivariable Cox regression analysis further solidified that TKI+chemotherapy was not an independent prognosticator for LRFS (HR = 1.32, 95% CI: 0.58-2.79; P = 0.547; Table 5).

**Table 5:** Univariable and multivariable Cox regressions of locoregional-free survival.

	**Univariable analysis**			**Multivariable analysis**		
	**HR**	**95% CI**	***P***	**HR**	**95% CI**	***P***
Age						
≤63	reference					
>63	0.54	0.28-1.04	0.065	0.61	0.20-1.07	0.070
Sex						
Male	reference					
Female	0.74	0.40-1.40	0.360	0.46	0.18-1.10	0.081
Smoking status						
Never smoker	reference					
Former smoker	0.76	0.32-1.81	0.531	0.45	0.11-1.33	0.132
Current smoker	2.78	0.64-12.01	0.172	1.26	0.12-5.44	0.815
ECOG						
0	reference					
1	0.63	0.33-1.22	0.169	0.55	0.23-1.31	0.175
T stage						
T1	reference					
T2	1.53	0.63-3.73	0.348	1.65	0.68-4.42	0.245
T3	1.18	0.37-3.74	0.778			
T4	2.31	0.88-6.04	0.089			
N stage						
N0	reference					
N1	2.05	0.13-32.86	0.613	4.18	0.17-113.51	0.374
N2	1.43	0.19-10.84	0.729			
N3	1.45	0.19-10.79	0.719			
AJCC stage						
IIIa	reference					
IIIb	1.07	0.51-2.21	0.863			
IIIc	1.57	0.60-4.07	0.356			
EGFR mutation						
Exon 19 deletion	reference					
L858R mutation	1.01	0.51-1.99	0.979	1.41	0.63-3.26	0.387
Other	0.75	0.18-3.20	0.699	1.10	0.16-5.07	0.915
Treatments						
TKI	reference					
TKI+chemotherapy	1.46	0.75-2.81	0.267	1.32	0.58-2.79	0.547

ECOG, Eastern Cooperative Oncology Group; AJCC, American Joint Committee on Cancer; TKI, tyrosine kinase inhibitor; EGFR, epidermal growth factor receptor; HR, hazard ratio; CI, confidence interval.

In the PSM cohort, the median LRFS for the TKI alone group was 75 months, compared to 49 months for the TKI+chemotherapy group (Figure 5B). The respective 1-year LRFS rates were 74.7% and 49.0%, while the 2-year LRFS rates were 37.4% and 36.8%.

### DMFS

**Figure 6:**
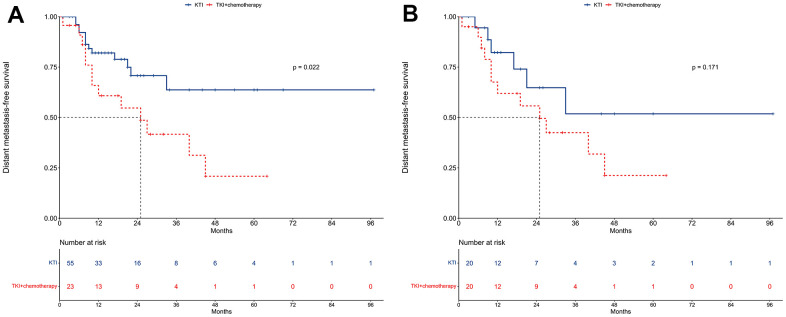
**Distant metastasis-free survival between TKI alone and TKI+chemotherapy groups.** (**A**) The unmatched cohort. (**B**) The propensity score matching cohort.

In the unmatched cohort, the median DMFS was not reached for the TKI alone group, while it was 25 months for the TKI+chemotherapy group (Figure 6A). The 1-year DMFS rates were 82.0% and 60.8%, and the 2-year DMFS rates were 70.7% and 54.7%, respectively for the two groups. The univariable regression analysis indicated a decreased DMFS with TKI+chemotherapy compared to TKI alone (HR = 2.39, 95% CI: 1.11-5.18; P = 0.022; Table 6). However, upon conducting a multivariable Cox regression analysis, TKI+chemotherapy did not emerge as an independent prognostic factor for DMFS (HR = 4.07, 95% CI: 0.97-6.22; P = 0.057; Table 6).

**Table 6:** Univariable and multivariable Cox regressions of distant metastasis-free survival.

	**Univariable analysis**			**Multivariable analysis**		
	**HR**	**95% CI**	***P***	**HR**	**95% CI**	***P***
Age						
≤63	reference					
>63	0.37	0.15-0.87	0.024	1.98	0.72-5.20	0.192
Sex						
Male	reference					
Female	1.20	0.55-2.63	0.639	1.33	0.42-4.02	0.644
Smoking status						
Never smoker	reference					
Former smoker	0.75	0.25-2.19	0.593	1.60	0.14-2.85	0.543
Current smoker	3.46	0.78-15.40	0.104	2.10	0.38-35.79	0.259
ECOG						
0	reference					
1	0.61	0.27-1.37	0.229	0.49	0.17-1.39	0.170
T stage						
T1	reference					
T2	1.31	0.48-3.56	0.596	1.51	0.50-4.40	0.482
T3	1.19	0.33-4.23	0.789			
T4	0.97	0.29-3.21	0.959			
N stage						
N0	reference					
N1	1.31	0.48-3.56	0.998			
N2	1.19	0.33-4.23	0.998			
N3	0.97	0.29-3.21	0.998			
AJCC stage						
IIIa	reference					
IIIb	1.00	0.42-2.36	0.995			
IIIc	0.68	0.18-2.56	0.568			
EGFR mutation						
Exon 19 deletion	reference					
L858R mutation	1.05	0.42-2.16	0.913	1.17	0.46-3.27	0.683
Other						
Treatments						
TKI	reference					
TKI+chemotherapy	2.39	1.11-5.18	0.022	4.07	0.97-6.22	0.057

ECOG, Eastern Cooperative Oncology Group; AJCC, American Joint Committee on Cancer; TKI, tyrosine kinase inhibitor; EGFR, epidermal growth factor receptor; HR, hazard ratio; CI, confidence interval.

In the PSM cohort, similar trends were observed with median DMFS not being reached for the TKI alone group and being 25 months for the TKI+chemotherapy group (Figure 6B). For the TKI alone and TKI+chemotherapy groups, the respective 1-year DMFS rates were 82.2% and 61.9%, while the respective 2-year DMFS rates were 64.7% and 55.7%.

### Adverse events

A comprehensive overview of the adverse events is presented in Table 7. The TKI+chemotherapy group was associated with a higher occurrence of hematological events compared to TKI alone group. Specifically, a higher incidence of leukopenia (Grade 1-2: 52.2% vs. 5.5%, P < 0.001), neutropenia (Grade 1-2: 56.5% vs. 5.5%, P < 0.001), anemia (Grade 1-2: 17.4% vs. 3.6%, P = 0.038), and thrombocytopenia (Grade 1-2: 13.0% vs. 0.0%, P = 0.006) was observed in the TKI+chemotherapy group.

**Table 7:** Adverse events between TKI alone and TKI+chemotherapy groups.

	**Grade 1-2**		***P***	**Grade 3-4**		***P***
	**TKI alone (n=55)**	**TKI+chemotherapy (n=23)**		**TKI alone (n=55)**	**TKI+chemotherapy (n=23)**	
Hematological events						
Leukopenia			<0.001			0.006
no	52 (94.5%)	11 (47.8%)		55 (100.0%)	20 (87.0%)	
yes	3 (5.5%)	12 (52.2%)		0 (0.0%)	3 (13.0%)	
Neutropenia			<0.001			0.027
no	52 (94.5%)	10 (43.5%)		55 (100.0%)	21 (91.3%)	
yes	3 (5.5%)	13 (56.5%)		0 (0.0%)	2 (8.7%)	
Anemia			0.038			0.295
no	53 (96.4%)	19 (82.6%)		55 (100.0%)	22 (95.7%)	
yes	2 (3.6%)	4 (17.4%)		0 (0.0%)	1 (4.3%)	
Thrombocytopenia			0.006			0.999
no	55 (100.0%)	20 (43.5%)		55 (100.0%)	23 (100.0%)	
yes	0 (0.0%)	3 (13.0%)		0 (0.0%)	0 (0.0%)	
Non-hematological events						
Liver dysfunction			0.540			0.999
no	45 (81.8%)	17 (73.9%)		55 (100.0%)	23 (100.0%)	
yes	10 (18.2%)	6 (26.1%)		0 (0.0%)	0 (0.0%)	
Rash or acne			0.805			0.999
no	29 (52.7%)	11 (47.8%)		51 (92.7%)	21 (91.3%)	
yes	26 (47.3%)	12 (52.2%)		4 (7.3%)	2 (8.7%)	
Diarrhea			0.805			0.999
no	33 (60.0%)	13 (56.5%)		53 (96.4%)	22 (95.7%)	
yes	22 (40.0%)	10 (43.5%)		2 (3.6%)	1 (4.3%)	
Vomiting			0.258			0.295
no	49 (89.1%)	18 (78.3%)		55 (100.0%)	22 (95.7%)	
yes	6 (10.9%)	5 (21.7%)		0 (0.0%)	1 (4.3%)	
Nail changes			0.999			0.999
no	47 (85.5%)	20 (87.0%)		54 (98.2%)	23 (100.0%)	
yes	8 (14.5%)	3 (13.0%)		1 (1.8%)	0 (0.0%)	

TKI, tyrosine kinase inhibitor.

Moreover, a higher prevalence of Grade 3-4 leukopenia (13.0% vs. 0.0%, P = 0.006) and neutropenia (8.7% vs. 0.0%, P = 0.027) was noted among patients receiving TKI+chemotherapy. In contrast, non-hematological events did not show any significant differences between the TKI alone and TKI plus chemotherapy groups (all P > 0.05).

## DISCUSSION

The findings of this study revealed two important insights. Firstly, it highlighted the prevailing trend in clinical practice where the majority of patients with unresectable stage III EGFR-mutated NSCLC were primarily treated with TKI monotherapy, as opposed to chemoradiotherapy. Secondly, our data suggested that the addition of chemotherapy to TKI therapy as an initial treatment strategy did not provide survival benefits over TKI alone.

The current standard of care for unresectable stage III EGFR-mutated NSCLC patients is CCRT [6–11]. Surprisingly, our study found that only a small proportion (4.5%) of patients received radiotherapy, while a significant majority (91.0%) were initially treated with TKI. There could be several explanations for this observation. One possible explanation was the evidence from various clinical trials that have demonstrated the efficacy of TKI as a standard treatment for stage IV EGFR-mutated NSCLC patients [18–24]. These trials had also included a subset of stage III patients, who achieved superior PFS with TKI monotherapy compared to CCRT [16, 17]. Another explanation could be the lack of difference in OS (HR = 0.71, 95% CI: 0.34-1.47) and cancer-specific survival (HR = 0.65, 95% CI: 0.31-1.35) between TKI monotherapy and CCRT, as reported in a previous study [25]. Additionally, the absence of radiation oncologists in defining treatment strategies could also contribute to the low utilization of radiotherapy [26].

While TKI monotherapy has shown better PFS than CCRT, it is crucial to recognize that it is not a curative treatment. TKI alone did not improve OS for stage III patients compared to stage IV patients [27]. Moreover, patients who experienced recurrences after CCRT had the option of salvage TKI therapy, which had shown significant OS improvement [28–30]. Therefore, recommending EGFR-TKI monotherapy as the initial treatment for all stage III patients may not be appropriate.

The FLAURA2 study had emphasized the improved PFS in stage IV patients receiving TKI plus chemotherapy [31, 32]. This raises the intriguing possibility of whether similar advantages could be observed in patients with stage III disease. Currently, however, there is a paucity of evidence addressing this question directly. In our study, we observed that the median PFS was 17.0 months in the TKI alone group and 11.0 months in the TKI+chemotherapy group, with no statistically significant difference between the two groups (HR = 1.56, 95% CI: 0.87-2.80; P = 0.134). This finding was consistent with several other studies that had reached similar conclusions [25, 29, 33, 34].

While these findings contribute valuable insights, it is crucial to interpret them with caution. A majority of participants in our study (89.7%) received first-generation TKIs, which had not demonstrated improved PFS when combined with chemotherapy in stage III patients [35]. It is noteworthy that third-generation TKIs have shown greater efficacy compared to their first-generation counterparts [18–20]. Therefore, there is a compelling rationale to investigate the therapeutic potential of combining third-generation TKIs with chemotherapy in stage III EGFR-mutated NSCLC patients.

It is important to acknowledge the limitations of our study. The retrospective design inherently results in potential confounders, such as age, sex, and T stage, being imbalanced between the treatment groups. Although we attempted to account for these factors through multivariable analysis and PSM, the possibility of unmeasured confounders remains. Moreover, the relatively small sample size of the TKI+chemotherapy group may have limited our ability to detect subtle differences in survival outcomes between the two treatment strategies.

In conclusion, our findings suggested that the addition of chemotherapy to TKI therapy did not improve survival outcomes in unresectable stage III EGFR-mutated NSCLC patients compared to TKI alone. However, given the limitations of our study, including its small sample size, these results should be interpreted cautiously. Larger prospective studies and clinical trials are essential to validate these findings and provide more definitive guidance on the optimal treatment approach for these patients.

## MATERIALS AND METHODS

### Patient selection

We conducted a comprehensive search to identify NSCLC patients at Guangxi Medical University Cancer Hospital between September 2012 and January 2022. The inclusion criteria for patient selection were as follows: (1) histologically or cytologically confirmed NSCLC, (2) EGFR testing performed and mutation confirmed, (3) definitive EGFR subtypes identified for the EGFR mutation, (4) staging according to the 8th edition of the American Joint Committee on Cancer (AJCC) criteria confirming stage III disease, and (5) presence of unresectable disease. Patients meeting any of the following criteria were excluded from the study: (1) those who did not receive any treatment, (2) patients who underwent surgery as their initial treatment modality, and (3) individuals with incomplete or missing clinical information.

### Endpoint

The primary endpoint of the study was progression-free survival (PFS). The secondary endpoints included overall survival (OS), locoregional-free survival (LRFS), and distant metastasis-free survival (DMFS).

### Treatment-related toxicities

Treatment-related toxicities were evaluated and categorized using the Common Toxicity Criteria for Adverse Events version 4.0 (CTCAE v4.0).

### Statistical analysis

The continuous factor of age was transformed to categorical factor according to the median value. Categorical variables were assessed using the χ^2^ test or Fisher’s exact test. To compare PFS, OS, LRFS, and DMFS across different treatment modalities, the Kaplan-Meier method coupled with log-rank test statistics was utilized. Univariable regression analysis was performed to pinpoint potential prognostic indicators. Furthermore, multivariable Cox regression analysis was conducted to identify independent prognostic factors after adjusting for variables. The results were presented in terms of hazard ratios (HRs) along with corresponding 95% confidence intervals (CIs).

To minimize the impact of selection bias when comparing outcomes between different treatment modalities, a matched case-control analysis via propensity score matching (PSM) was executed. During the calculation of propensity scores, one-to-one matching without replacement was achieved within a logistic regression model. The nearest-neighbor matching algorithm based on the propensity score was applied for factors with a caliper of 0.8 on the logistic regression model.

The statistical analyses for this study were carried out using SPSS Statistics Version 26.0 software (IBM Co., Armonk, NY, USA) and R software (version 4.2.1). A two-tailed P-value of less than 0.05 was deemed statistically significant.

### Data availability statement

The data generated or analyzed during this study are available from the corresponding author upon reasonable request.
